# Risk analysis of the association between EASIX and all-cause mortality in critical ill patients with atrial fibrillation: a retrospective study from MIMIC-IV database

**DOI:** 10.1186/s40001-025-02621-4

**Published:** 2025-04-29

**Authors:** Yu Xia, Anfeng Liang, Mei Wang, Jianlin Zhang

**Affiliations:** 1 Department of Burn and Trauma Medicine, First Naval Hospital of Southern Theater Command, Zhanjiang, China; 2 Trauma Center, Jinling Hospital, Medical School of Nanjing University, Nanjing, China; 3 Department of Emergency Medicine, The First Affiliated Hospital of Anhui Medical University, Hefei, China

**Keywords:** Atrial fibrillation, Endothelial activation and stress index (EASIX), Endothelial dysfunction, MIMIC-IV, All-cause mortality

## Abstract

**Background:**

The Endothelial Activation and Stress Index (EASIX) is a recognized marker of vascular endothelial health but has limited application in patients with atrial fibrillation (AF). This study aimed to explore the association between EASIX and prognosis in critically ill patients with AF.

**Methods:**

The patient’s data were extracted from Medical Information Mart for Intensive Care IV(MIMIC-IV) database. EASIX was calculated as lactate dehydrogenase (U/L) × creatinine (mg/dL)/platelets (10^9^ cells/L) and log2-transformed for statistical analysis. The Boruta algorithm and Least Absolute Shrinkage and Selection Operator (Lasso) Regression were used for feature selection. Multivariable logistic regression and Cox proportional hazard models were employed to assess EASIX as a risk factor, with nonlinear relationships evaluated using restricted cubic spline curves. The area under the receiver operating characteristic curve (AUC) was utilized to compare the predictive performance of EASIX with the Sequential Organ Failure Assessment (SOFA) score and the CHA₂DS₂–VASc score. Furthermore, Kaplan–Meier survival analysis based on EASIX quartiles (with Q1 as the reference) and stratified analyses were conducted to further explore these associations.

**Results:**

A total of 4896 patients with complete data were included. In-hospital, 28-day, and 365-day all-cause mortality rates were26.04%, 29.25%, and 49.75%, respectively. The median EASIX was 5.64 (4.56, 6.84). Higher EASIX was significantly associated with increased in-hospital, short-term, and long-term all-cause mortality after multivariable adjustment. Patients in quartiles Q2, Q3, and Q4 had significantly higher mortality than those in Q1, showing a clear trend. Kaplan–Meier analysis confirmed that patients with higher EASIX scores had significantly lower survival. The AUC showed that the performance of EASIX in predicting both short-term and long-term all-cause mortality was comparable to the SOFA and higher than the CHA₂DS₂–VASc score. Stratified analyses indicated that the association remained robust across subgroups, accounting for various underlying conditions and hospital interventions.

**Conclusions:**

EASIX is a reliable predictor of both short- and long-term mortality in critically ill patients with AF. Future prospective studies are necessary to confirm its broader applicability in other populations.

**Supplementary Information:**

The online version contains supplementary material available at 10.1186/s40001-025-02621-4.

## Introduction

Atrial fibrillation (AF) is the most prevalent sustained cardiac arrhythmia, with a lifetime risk of approximately 25% in the general population [1]^.^ Its prevalence increases significantly with age, reaching 10–12% in individuals aged 80 years and older [2]. Globally, the prevalence of AF is estimated to affect 33.5 million individuals, constituting 2.5–3.5% of the overall population in numerous countries [3]. AF has been demonstrated to be strongly associated with elevated risks of all-cause mortality, heart failure, hospitalization, and thromboembolic events [4].

A plethora of studies have revealed a robust link between endothelial dysfunction (ED) and the development and progression of AF. The imbalance between inducible nitric oxide synthase (iNOS) and endothelial nitric oxide synthase (eNOS) following endothelial injury has been demonstrated to promote inflammation and myocardial fibrosis. In addition, elevated levels of von Willebrand factor (vWF) [5] have been shown to contribute to the formation of atrial thrombi in patients with AF. Furthermore, the hemodynamic alterations commonly observed in AF patients exacerbate endothelial damage and induce a hypercoagulable state [7], which significantly increases the mortality risk in this population.

The Endothelial Activation and Stress Index (EASIX) has recently emerged as a novel biomarker for assessing ED [9]. It is calculated using the following formula: serum lactate dehydrogenase (LDH) level (U/L) × creatinine level (mg/dL)/platelet count (10^9^/L). Researchers have identified a strong association between EASIX and endothelial activation markers, including interleukin-18, chemokine–X–C-ligand 8, insulin-like growth factor-1, and suppressor of tumorigenicity-2 [10, 11]. EASIX has also been used to predict mortality in patients with hematologic malignancies following allogeneic stem cell transplantation (allo-SCT) [12]. Furthermore, EASIX has been validated as a prognostic marker for mortality in various conditions, including multiple myeloma [13], COVID-19 [14], small cell lung cancer [15], urothelial carcinoma [16], traumatic brain injury [17], and sepsis [18].

However, the potential of EASIX as a prognostic marker in patients with AF, particularly those who are critically ill, remains underexplored. Unlike traditional scoring systems, such as congestive heart failure, hypertension, age, diabetes mellitus, stroke, vascular disease, and sex category (CHA₂DS₂–VASc) score [19], which primarily predict thromboembolic events, or Sequential Organ Failure Assessment (SOFA) [20],which is used to assess the severity of organ dysfunction in critically ill patients, EASIX offers a unique advantage by directly reflecting ED, a crucial factor in AF pathogenesis. Given the growing use of EASIX in other diseases and its proven role in outcome prediction, this study aims to assess its prognostic value as a mortality predictor in critically ill patients with AF.

## Materials and methods

### Data sources and extraction

This study utilized data from the Medical Information Mart for Intensive Care-IV(MIMIC-IV) database, which consists of electronic health records of critically ill patients from the Beth Israel Deaconess Medical Center, a single-center institution. The MIMIC-IV database is an open-access resource, and access to the data was granted to one of the authors upon completion of CITI training and approval from PhysioNet (record ID: 59,051,976), with authorization from the relevant institutional authorities. The study team did not participate in data collection; all data were obtained from the MIMIC-IV database and analyzed following the database's established usage guidelines.

The data were extracted from the MIMIC-IV(version 3.0) database using the pgAdmin PostgreSQL tools (version 1.22.1) and comprised demographic information, laboratory results, vital signs, comorbidities, medications and interventions, evaluation scores, and endpoints.

In accordance with prior research [21], the diagnoses of AF, heart failure(HF), myocardial infarction (MI), malignant tumor, chronic obstructive pulmonary disease (COPD), hypertension, stroke, diabetes, hyperlipidemia, and chronic kidney disease (CKD) in this study were based on diagnoses using codes from both the International Classification of Diseases, Ninth Revision (ICD-9) and Tenth Revision (ICD-10). The ICD-9 and ICD-10 codes for all diseases are presented in Supplementary Table S1.

**Fig. 1:**
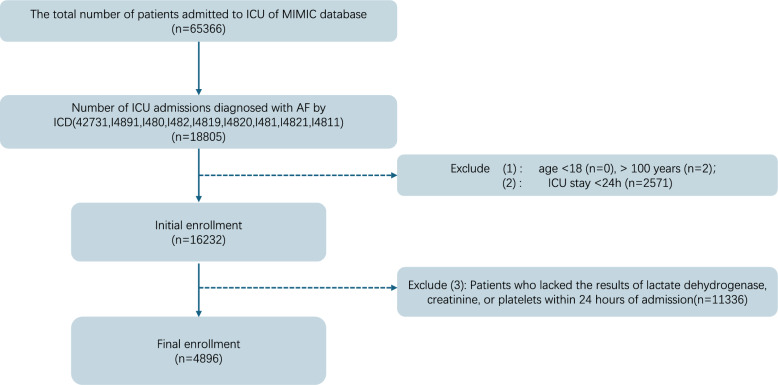
Selection flowchart of AF patients from the MIMIC-IV database

A total of 65,366 patients were admitted to the ICU, of whom 18,805 were diagnosed with AF. The exclusion criteria included: (1) patients aged under 18 or over 100 years; (2) patients with an ICU stay of less than 24 h; and (3) patients who lacked lactate dehydrogenase, creatinine, or platelet data within 24 h of admission. After applying these criteria, the final cohort consisted of 4896 patients (Fig. 1).Variables with a missing data rate exceeding 20% were excluded, and multiple imputations were performed for other incomplete data. The multivariate imputation by chained equations (MICE) method was employed using the "mice" package in R, and the complete data set was ultimately returned based on the standard errors and *P* values of the model.

### Feature selection

We aimed to identify whether EASIX is associated with outcomes through feature selection while simultaneously selecting covariates for inclusion in the subsequent multivariable adjustment model. To achieve this, we employed both the Boruta algorithm and Lasso regression. (1) Boruta algorithm: a machine learning algorithm based on Random Forest, selects features by comparing their importance to that of randomly permuted "shadow" features. Feature importance, measured by metrics like the Gini index or mean decrease in accuracy, reflects their contribution to the model's predictive performance. Features with higher Z-scores (calculated as the difference between the feature’s importance and the mean importance of shadow features, divided by their standard deviation) are retained, while those with lower importance are discarded [22]; (2) Lasso regression: a penalized regression technique that selects relevant features by shrinking the coefficients of less important variables to zero. The strength of the penalty is determined by the parameter Lambda (λ), which is chosen to minimize cross-validation error. This process helps prevent overfitting while retaining the most significant predictors. The optimal λmin strikes a balance between model complexity and predictive accuracy, ensuring both robust performance and interpretability [23]. The final model will incorporate variables selected by both algorithms, ensuring the predictors strongly associated with the outcome while mitigating the risk of multicollinearity.

### Definition of exposure variables and endpoint events

The EASIX was calculated based on the formula: lactate dehydrogenase (U/L) × creatinine (mg/dL)/platelets (10^9^ cells/L) and log2 conversion for statistical analysis (all EASIX shown in this article are log2 transformed) [11].

The CHA2DS2–VASc score [gender (female), 1 point; age: ≥ 75 years, 2 points; age: 65–74 years, 1 point; previous stroke, 2 points; congestive heart failure, 1 point; hypertension, 1 point; diabetes, 1 point; vascular disease or myocardial infarction, 1 point] is widely used to stratify AF patients according to their risk of stroke, with higher scores indicating a greater risk [19]. Based on the comorbidity data extracted in this study, we calculated the CHA₂DS₂–VASc score for each patient. Given its established role in predicting thromboembolic events, CHA₂DS₂–VASc was incorporated into this study both as a potential confounder and for comparison with EASIX.

The primary endpoint of the present study was in-hospital mortality, with secondary endpoints including 28-day and 365-day mortality.

### Statistical analysis

As this study is a retrospective analysis, no sample size calculations were performed. Continuous variables were first assessed for normality. For normally distributed data, Student’s *t* tests were conducted, and results are presented as mean ± standard deviation (SD). Non-normally distributed data were analyzed using the Wilcoxon rank-sum test, with results expressed as median and interquartile range (IQR). Categorical variables were analyzed using chi-square or Fisher’s exact tests, with results reported as absolute numbers and percentages. To evaluate the association between the EASIX and the risk of in-hospital mortality, as well as 28-day and 365-day mortality, univariable and multivariable logistic regression or Cox regression analyses were employed. Model 1 included only the EASIX index without any adjustments. Model 2 adjusted for gender, age, and race, while Model 3 was fully adjusted, incorporating confounders identified through the Boruta algorithm, Lasso regression and clinical expertise. The study population was categorized into four groups based on EASIX quartiles: (Q1: < 4.56, Q2: 4.56–5.64, Q3: 5.64–6.84, Q4: > 6.84). Kaplan–Meier survival analysis was conducted according to these quartiles, with the log-rank test used to assess differences between groups. In addition, a four-knotted multivariate restricted cubic spline (RCS) regression analysis was performed to explore potential nonlinear relationships between EASIX and the outcomes of interest. The area under the ROC curve (AUC) was used to assess and compare the prognostic potential of EASIX and other established scores in predicting in-hospital mortality, 28-day mortality, and 365-day mortality. Stratified analyses were also conducted to verify the robustness of the findings. Statistical analyses were carried out using R Studio (version R4.4.1, R Foundation for Statistical Computing, Austria), and a two-sided *p* value of < 0.05 was considered statistically significant.

## Results

### Baseline characteristics of study individuals

The study included 4896 patients with AF in the MIMIC-IV database, with 2871(58.64%) of the cohort being male. Of the total number of patients, 1275 (26.04%) died during their hospital stay, while the remaining 3621 survived. During follow-up, 1432 patients (29.25%) died within 28 days, and 2436 patients (49.75%) died within 1 year. A statistically significant difference was observed in age between hospital survivors and non-survivors (*p* < 0.001). The systolic blood pressure (*p* < 0.001), diastolic blood pressure (*p* < 0.001)and SpO2 (*p* < 0.024) were both significantly lower in non-survivors, while the heart rate (*p* < 0.001) and respiratory rate (*p* < 0.001) were significantly higher in survivors. With regard to the results of laboratory tests, with the exception of sodium (*p* = 0.482), which demonstrated no significant differences, the majority of indicators exhibited marked disparities between the two groups. It is noteworthy that lactate dehydrogenase (*p* < 0.001) and serum creatinine (*p* < 0.001) levels were higher in non-survivors, whereas platelet levels (*p* < 0.001) exhibited the opposite trend. This resulted in a significantly higher EASIX in non-survivors (*p* < 0.001). In addition, non-survivors exhibited more severe conditions, as evidenced by higher scores on the SOFA, OASIS, APS III, SAPS II, and Charlson index, along with a lower GCS (*p* < 0.001). However, there was no significant difference in CHA_2_DS_2_–VASc scores between the two groups (*p* = 0.529). In addition, non-survivors had a significantly higher prevalence of sepsis (*p* < 0.001) and acute kidney injury (AKI) (*p* < 0.001) compared to survivors, and experienced a shorter hospitalization duration (*p* < 0.001) but a prolonged ICU stay (*p* < 0.001) (Table 1).

**Table 1:** Baseline characteristics of study population

Variables	Total (*n* = 4896)	Survivors (*n* = 3621)	None-survivors (*n* = 1275)	*P*
Demographics				
Age, years	75.00 (66.00, 83.00)	75.00 (66.00, 83.00)	76.00 (68.00, 84.00)	0.006
Male Gender, *n* (%)	2871 (58.64)	2118 (58.49)	753 (59.06)	0.724
Race, White, *n* (%)	3093 (63.17)	2300 (63.52)	793 (62.20)	0.400
Weight, Kg	79.80 (66.80, 95.60)	80.00 (67.00, 95.50)	79.00 (66.55, 96.05)	0.386
Past history, *n* (%)				
Hypertension	1676 (34.23)	1292 (35.68)	384 (30.12)	< 0.001
Heart Failure	2656 (54.25)	1966 (54.29)	690 (54.12)	0.913
Myocardial infarction	725 (14.81)	509 (14.06)	216 (16.94)	0.013
Malignant tumor	940 (19.20)	676 (18.67)	264 (20.71)	0.112
CKD	1523 (31.11)	1087 (30.02)	436 (34.20)	0.006
COPD	643 (13.13)	461 (12.73)	182 (14.27)	0.161
Hyperlipidemia	2189 (44.71)	1681 (46.42)	508 (39.84)	< 0.001
Stroke	538 (10.99)	420 (11.60)	118 (9.25)	0.021
Diabetes	1740 (35.54)	1274 (35.18)	466 (36.55)	0.381
Laboratory data				
WBC(10^9^/L)	11.20 (8.10, 16.00)	10.77 (7.90, 15.10)	12.75 (8.70, 18.60)	< 0.001
RBC(10^9^/L)	3.42 (2.94, 3.98)	3.48 (3.01, 4.02)	3.28 (2.80, 3.83)	< 0.001
Platelet(10^9^/L)	182.00 (127.64, 250.00)	185.00 (134.00, 251.33)	173.00 (109.07, 247.00)	< 0.001
Hemoglobin(g/dL)	10.15 (8.70, 11.78)	10.30 (8.90, 11.90)	9.67 (8.35, 11.30)	< 0.001
RDW(%)	15.27 (14.07, 17.15)	15.00 (13.90, 16.73)	16.10 (14.60, 18.12)	< 0.001
Hct(%)	31.27 (27.15, 36.10)	31.65 (27.50, 36.43)	30.06 (26.23, 35.31)	< 0.001
Sodium(mmol/L)	138.20 (135.00, 141.00)	138.25 (135.33, 141.00)	138.00 (134.50, 141.67)	0.482
Potassium(mmol/L)	4.20 (3.85, 4.60)	4.15 (3.83, 4.58)	4.30 (3.90, 4.79)	< 0.001
Calcium(mmol/L)	8.35 (7.87, 8.80)	8.38 (7.90, 8.83)	8.27 (7.75, 8.75)	< 0.001
Chloride(mmol/L)	103.00 (98.67, 107.00)	103.00 (99.00, 107.00)	102.33 (97.63, 107.00)	0.010
Glucose(mg/dL)	131.12 (109.00, 166.76)	128.50 (108.00, 161.00)	140.75 (113.88, 180.74)	< 0.001
Anion gap(mmol/L)	14.50 (12.33, 17.00)	14.00 (12.00, 16.67)	16.00 (13.33, 19.33)	< 0.001
PT(s)	15.53 (13.40, 20.80)	15.10 (13.18, 19.40)	17.25 (14.09, 24.93)	< 0.001
PTT(s)	34.30 (29.00, 47.50)	33.60 (28.73, 45.40)	36.86 (30.15, 52.69)	< 0.001
INR	1.40 (1.20, 1.90)	1.40 (1.20, 1.80)	1.60 (1.30, 2.30)	< 0.001
Bilirubin(mg/dL)	0.70 (0.40, 1.30)	0.70 (0.40, 1.20)	0.85 (0.50, 1.80)	< 0.001
ALT(U/L)	26.00 (16.00, 60.00)	24.50 (15.00, 52.00)	32.00 (17.00, 88.41)	< 0.001
AST(U/L)	39.00 (23.00, 89.00)	36.00 (22.00, 75.50)	52.00 (27.50, 145.25)	< 0.001
Urea nitrogen(mg/dL)	28.67 (18.00, 46.67)	25.50 (17.00, 42.75)	38.00 (25.00, 58.55)	< 0.001
Serum creatinine(mg/dL)	1.30 (0.90, 2.13)	1.20 (0.85, 1.90)	1.65 (1.10, 2.77)	< 0.001
LDH(U/L)	287.00 (215.00, 427.62)	268.00 (206.00, 379.00)	360.00 (255.00, 598.00)	< 0.001
Vital signs				
Heart Rate(bpm)	86.07 (74.57, 99.37)	84.82 (73.63, 97.74)	90.00 (78.06, 103.58)	< 0.001
Systolic blood pressure(mmHg)	111.03 (102.00, 123.87)	112.50 (102.97, 125.67)	107.24 (99.61, 118.42)	< 0.001
Diastolic blood pressure(mmHg)	62.96 (56.20, 70.43)	63.28 (56.60, 70.90)	61.67 (55.25, 69.09)	< 0.001
Mean blood pressure(mmHg)	75.28 (68.91, 83.55)	75.96 (69.43, 84.33)	73.70 (67.74, 81.06)	< 0.001
Respiratory rate(bpm)	19.76 (17.40, 22.63)	19.45 (17.16, 22.11)	20.92 (18.30, 24.09)	< 0.001
SpO2(%)	96.75 (95.27, 98.13)	96.79 (95.36, 98.10)	96.64 (95.00, 98.21)	0.024
Temperature (°C)	36.77 (36.56, 37.04)	36.78 (36.58, 37.02)	36.75 (36.51, 37.10)	0.128
Medication, *n* (%)				
Glucocorticoids	1292 (26.39)	837 (23.12)	455 (35.69)	< 0.001
ARB/ACEI	1131 (23.10)	1049 (28.97)	82 (6.43)	< 0.001
Immunosuppressant	132 (2.70)	98 (2.71)	34 (2.67)	0.940
Aspirin	2336 (47.71)	1884 (52.03)	452 (35.45)	< 0.001
Statins	639 (13.05)	534 (14.75)	105 (8.24)	< 0.001
Beta blocker	3537 (72.24)	2802 (77.38)	735 (57.65)	< 0.001
Clopidogrel	96 (1.96)	68 (1.88)	28 (2.20)	0.481
Dipyridamo	47 (0.96)	44 (1.22)	3 (0.24)	0.002
Warfarin	939 (19.18)	847 (23.39)	92 (7.22)	< 0.001
Amiodarone	1242 (25.37)	798 (22.04)	444 (34.82)	< 0.001
Digitalis	399 (8.15)	271 (7.48)	128 (10.04)	0.004
Diuretics	3302 (67.44)	2454 (67.77)	848 (66.51)	0.408
Norepinephrine	2206 (45.06)	1276 (35.24)	930 (72.94)	< 0.001
Phenylephrine	1795 (36.66)	1116 (30.82)	679 (53.25)	< 0.001
Vasopressin	1107 (22.61)	474 (13.09)	633 (49.65)	< 0.001
Dopamine	282 (5.76)	149 (4.11)	133 (10.43)	< 0.001
Dobutamine	332 (6.78)	168 (4.64)	164 (12.86)	< 0.001
Epinephrine	451 (9.21)	251 (6.93)	200 (15.69)	< 0.001
Intervention, *n* (%)				
Ventilation	4099 (83.72)	2968 (81.97)	1131 (88.71)	< 0.001
CRRT	502 (10.25)	223 (6.16)	279 (21.88)	< 0.001
Length of stay (LOS), days				
LOS hospital	10.02 (5.94, 17.46)	10.50 (6.41, 17.78)	8.99 (4.22, 16.70)	< 0.001
LOS ICU	3.32 (1.94, 6.64)	3.06 (1.87, 5.73)	4.59 (2.27, 9.02)	< 0.001
Evaluation scores				
SOFA	6.00 (3.00, 9.00)	5.00 (3.00, 8.00)	8.00 (5.00, 11.00)	< 0.001
OASIS	34.00 (29.00, 41.00)	33.00 (28.00, 39.00)	39.00 (33.00, 45.00)	< 0.001
APS III	51.00 (39.00, 67.00)	47.00 (36.00, 60.00)	66.00 (52.00, 84.00)	< 0.001
GCS	15.00 (14.00, 15.00)	15.00 (14.00, 15.00)	15.00 (13.00, 15.00)	< 0.001
SAPS II	42.00 (34.00, 52.00)	40.00 (33.00, 49.00)	51.00 (42.00, 62.00)	< 0.001
Charlson	7.00 (5.00, 8.00)	6.00 (5.00, 8.00)	7.00 (5.00, 9.00)	< 0.001
CHA2DS2 VASc	3.00 (2.00, 4.00)	3.00 (2.00, 4.00)	3.00 (2.00, 4.00)	0.529
EASIX	5.64 (4.56, 6.84)	5.38 (4.37, 6.46)	6.57 (5.35, 7.93)	< 0.001
Events, *\* (%)				
AKI	4181 (85.40)	2979 (82.27)	1202 (94.27)	< 0.001
Sepsis	3298 (67.36)	2221 (61.34)	1077 (84.47)	< 0.001
Death Within Hosp 28 days	1432 (29.25)	288 (7.95)	1144 (89.73)	< 0.001
Death Within Hosp 90 days	1933 (39.48)	667 (18.42)	1266 (99.29)	< 0.001
Death Within Hosp 180 days	2180 (44.53)	905 (24.99)	1275 (100.00)	< 0.001
Death Within Hosp 365 days	2436 (49.75)	1161 (32.06)	1275 (100.00)	< 0.001

Data are means ± SD, median (interquartile range), or n (%)

WBC, white blood cell; RBC, red blood cell; RDW, red cell distribution width; Hct, hematocrit; PT, prothrombin time; PTT, partial prothrombin time; INR, international normalized ratio; ALT, alanine aminotransferase; AST, aspartate aminotransferase; LDH, lactate dehydrogenase; SpO2:oxyhemoglobin saturation; ACEIs/ARBs, angiotensin-converting enzyme inhibitors/angiotensin receptor blockers; CRRT, continuous renal replacement therapy; ICU, intensive care unit; SOFA, sequential organ failure assessment; APS III:SAPS II, simplified acute physiology score; OASIS, Oxford acute severity of illness score**;** GCS, Glasgow coma scale; Charlson, Charlson comorbidity index; EASIX, endothelial activation and stress index; AKI, acute kidney injury

### Feature selection

**Fig. 2:**
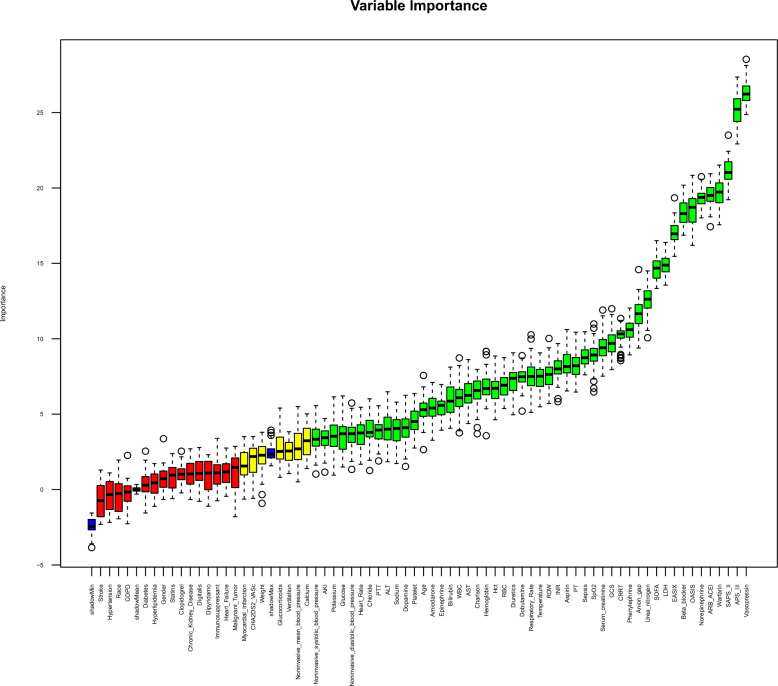
Boruta algorithm conducted the feature selection for the relationship between EASIX and in-hospital mortality. The horizontal axis shows the name of each variable, while the vertical axis represents the *Z* value of each variable. The box plot depicts the *Z* value of each variable in the model calculation, with green boxes representing important variables, yellow representing tentative attributes, and red representing unimportant variables

**Fig. 3:**
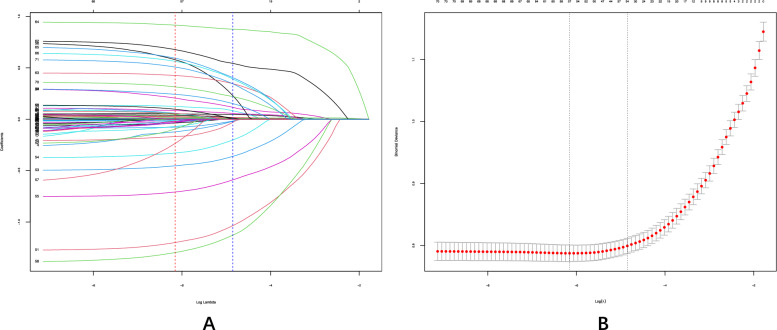
Lasso regression conducted the feature selection for the relationship between EASIX and in-hospital mortality. **A** Variation characteristics of the coefficients of variables as the regularization parameter λ changes. The plot shows how the coefficients shrink towards zero with increasing λ, highlighting the importance of each variable; **B** selection process of the optimal value of the regularization parameter λ in the Lasso regression model, determined through cross-validation. The plot illustrates the relationship between the mean cross-validation error and log (λ). The dashed vertical lines indicate two key values of λ: the value that minimizes the mean cross-validation error (λ_min) and the largest value of λ within one standard error of the minimum (λ_1se), used for model selection

As indicated by the Boruta algorithm, 49 of the 71 variables most strongly associated with in-hospital mortality were confirmed (Fig. 2, Supplementary Table S2). In addition, using Lasso regression, we identified 57 highly relevant variables while optimizing the lambda to minimize multicollinearity (Fig. 3, Supplementary Table S2). The intersection of the Boruta-selected variables and those selected by Lasso resulted in 38 variables being retained for further analysis, which were considered to have a significant impact on in-hospital mortality in patients with atrial fibrillation. Notably, EASIX was retained in both selection methods, highlighting its relevance across both approaches. Considering both clinical significance and the need to mitigate multicollinearity, we retained not only the intersection variables but also included additional factors such as gender, race, CKD, COPD and the CHA_2_DS_2_–VASc score as correction factors. Ultimately, 45 variables were incorporated into the fully adjusted model.

### Association between EASIX and in-hospital mortality

To comprehensively assess the role of EASIX, we analyzed it both as a continuous variable and categorized into quartiles. Participants were grouped by the EASIX quartiles at admission (Q1: < 4.56, Q2: 4.56–5.64, Q3: 5.64–6.84, and Q4: > 6.84) and their baseline characteristics are summarized in Supplementary Table S3.

**Fig. 4:**
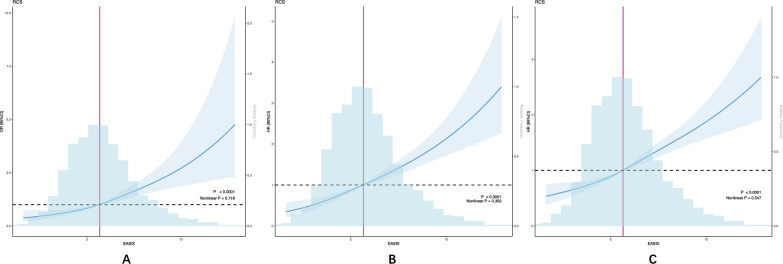
Restricted cubic spline regression analysis of EASIX with all-cause mortality. Restricted cubic spline regression analysis of EASIX with in hospital **A** 28-day, **B** 365-day **C** all-cause mortality

**Fig. 5:**
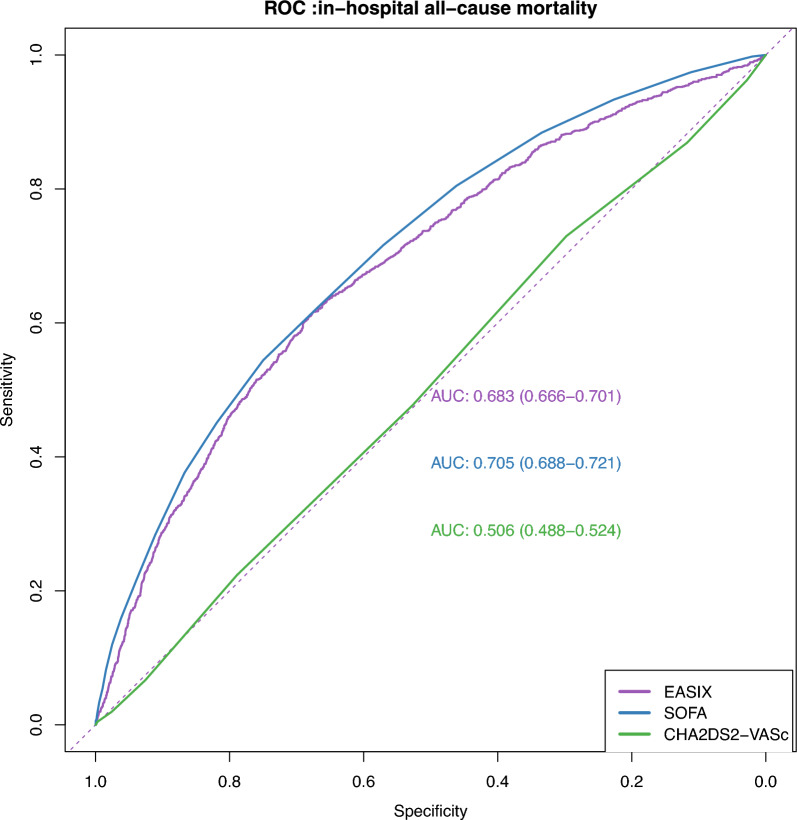
ROC curves for predicting in-hospital all-cause mortality

The results from the multivariable logistic regression analysis (Table 2) indicated that a higher EASIX was significantly associated with an increased risk of in-hospital mortality (OR 1.28, 95% confidence interval [CI] 1.19–1.37), after adjusting for all factors identified through Boruta analysis, Lasso regression and clinical judgment. When comparing to the lowest quartile of EASIX (Q1) as a reference (Table 2, P for trend < 0.001), the odds of in-hospital death increased in Q2 (OR 1.76, 95% CI 1.34–2.32), Q3 (OR 1.94, 95% CI 1.45–2.60), and Q4 (OR 3.08, 95% CI 2.19–4.33). Furthermore, no evidence of a nonlinear relationship between EASIX and in-hospital mortality was found in the RCS model (Nonlinear *P* = 0.718) (Fig. 4). We evaluated the predictive performance of EASIX, SOFA, and CHA₂DS₂–VASc scores for in-hospital mortality using ROC analysis. The AUCs were 0.683 (95% CI 0.666–0.701) for EASIX, 0.705 (95% CI 0.688–0.721) for SOFA, and 0.506 (95% CI 0.488–0.524) for CHA₂DS₂–VASc. Delong's test indicated that EASIX had a slightly lower AUC than SOFA (*P* = 0.016) but was significantly higher than CHA₂DS₂–VASc (*P* < 0.001) (Fig. 5).

**Table 2:** Associations of EASIX with in hospital mortality

	Model 1	Model 2	Model 3
In hospital mortality			
continuous			
Per 1-unit increment	1.41(1.36,1.46)***	1.42(1.36,1.47)***	1.28(1.19,1.37) ***
Categorical			
Q1(< 4.56)	Ref.	Ref.	Ref.
Q2(4.56–5.64)	1.76(1.42,2.20)***	1.75(1.40,2.18)***	1.76(1.34,2.32)***
Q3(5.64–6.84)	2.68 (2.17,3.32)***	2.67 (2.16,3.31)***	1.94(1.45,2.60)***
Q4(> 6.84)	5.84(4.77,7.19)***	5.93(4.83,7.30)***	3.08(2.19,4.33)***
P for trend	< 0.001	< 0.001	< 0.001

*P* value *P < 0.05 **P < 0.01 ***P < 0.001

Model 1 Univariate model

Model 2 adjusted for Age, Gender, Race

Model 3 adjusted for Age, Gender, Race, CKD, COPD, CHA2DS2–VASc score, and features confirmed by both Lasso and Boruta algorithms

### Association between EASIX and both 28-day and 365-day mortality

The association between EASIX and both 28-day and 365-day mortality was evaluated using Cox proportional hazards models. Multivariable Cox regression analysis revealed a significant correlation between elevated EASIX and increased risk of 28-day mortality (HR 1.21, 95% CI 1.16–1.26, *P* < 0.001). Compared to the lowest EASIX quartile (Q1, reference group, *P* for trend < 0.001), the hazard ratios (HRs) for 28-day mortality were notably higher in Q2 (HR 1.55, 95% CI 1.28–1.88), Q3 (HR 1.63, 95% CI 1.33–1.99), and Q4 (HR 2.36, 95% CI 1.88–2.96).A similar pattern was observed for 365-day mortality, with higher EASIX levels significantly correlating with increased risk of death at 1 year. Multivariable Cox regression confirmed that EASIX independently predicted 365-day mortality, where each 1-point rise in EASIX was associated with a 1.16-fold increased risk of death (HR 1.16, 95% CI 1.12–1.21) after adjusting for confounding variables. Furthermore, patients in the Q4, Q3, and Q2 quartiles had 1.39, 1.54, and 1.98 times the risk of 365-day mortality, respectively, compared to those in the lowest quartile (Q1) (Table 3). No evidence of a nonlinear relationship between EASIX and either 28-day (*P* = 0.302) or 365-day mortality (*P* = 0.547) was detected using restricted cubic spline (RCS) modeling (Fig. 4).

**Table 3:** Associations of EASIX with 28-day mortality and 365-day mortality

	Model 1	Model 2	Model 3
All-cause mortality within 28 days			
continuous			
Per 1-unit increment	1.27 (1.24, 1.30)***	1.29(1.26, 1.32)***	1.21(1.16, 1.26)***
categorical			
Q1(< 4.56)	Ref.	Ref.	Ref.
Q2(4.56–5.64) Q3(5.64–6.84)	1.63(1.36, 1.96) *** 2.07(1.74, 2.47)***	1.57(1.31, 1.88)*** 2.01(1.69, 2.40)***	1.55(1.28, 1.88)*** 1.63(1.33, 1.99) ***
Q4(> 6.84)	3.84(3.26, 4.53)***	3.89(3.30, 4.59)***	2.36(1.88, 2.96)***
Group trend	P < 0.001	P < 0.001	P < 0.001
All-cause mortality within 365 days			
continuous			
Per 1-unit increment	1.22 (1.19, 1.24) ***	1.24(1.21, 1.26)***	1.16(1.12, 1.21)***
categorical			
Q1(< 4.56)	Ref.	Ref.	Ref.
Q2(4.56–5.64)	1.45(1.28, 1.65) ***	1.40(1.23, 1.59)***	1.39(1.21, 1.59)***
Q3(5.64–6.84)	1.93(1.70, 2.18) ***	1.87(1.65, 2.12)***	1.54(1.34, 1.79)***
Q4(> 6.84)	3.01(2.67, 3.39) ***	3.06 (2.71, 3.45)***	1.98(1.67, 2.35)***
Group trend	*P* < 0.001	*P* < 0.001	*P* < 0.001

*P* value *P < 0.05 **P < 0.01 ***P < 0.001

Model 1 Univariate model

Model 2 adjusted for Age, Gender, Race

Model 3 adjusted for Age, Gender, Race, CKD, COPD, CHA2DS2–VASc score, and features confirmed by both Lasso and Boruta algorithms

ROC analysis showed that for 28-day mortality prediction, EASIX (AUC 0.664, 95% CI 0.647–0.681) was not significantly different from SOFA (AUC 0.678, 95% CI 0.661–0.694; *P* = 0.117) but was significantly higher than CHA₂DS₂–VASc (AUC 0.540, 95% CI 0.523–0.557; *P* < 0.001). Similar findings were observed for 365-day mortality, where EASIX (AUC 0.649, 95% CI 0.633–0.664) showed no significant difference from SOFA (AUC 0.641, 95% CI 0.626–0.656; *P* = 0.334) but remained significantly superior to CHA₂DS₂–VASc (AUC 0.558, 95% CI 0.542–0.574; *P* < 0.001) (Supplementary Fig. S1).

**Fig. 6:**
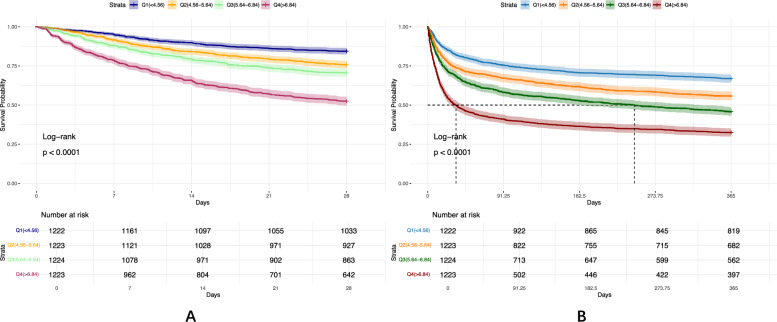
Kaplan–Meier survival analysis curves for all-cause mortality. Kaplan–Meier curves and cumulative incidence of 28-day (**A**) and 365-day (**B**) all-cause mortality stratified by EASIX

Kaplan–Meier survival analysis stratified by EASIX quartiles revealed significant differences in both 28-day and 365-day mortality across quartiles (log-rank *P* < 0.0001), with the highest EASIX group showing the poorest survival outcomes (Fig. 6). Corrected pairwise comparisons indicated significant differences between the four groups, both for 28-day mortality and 365-day mortality (Bonferroni *P* < 0.05).

### Stratified analyses

To further investigate whether the associations between EASIX and in-hospital, 28-day, and 365-day all-cause mortality held across various conditions, subgroup analyses were performed based on age, gender, race, hypertension, HF, MI, malignant tumors, CKD, COPD hyperlipidemia, stroke, and diabetes while adjusting for medications and interventions that are shown in the Table 1.

**Fig. 7:**
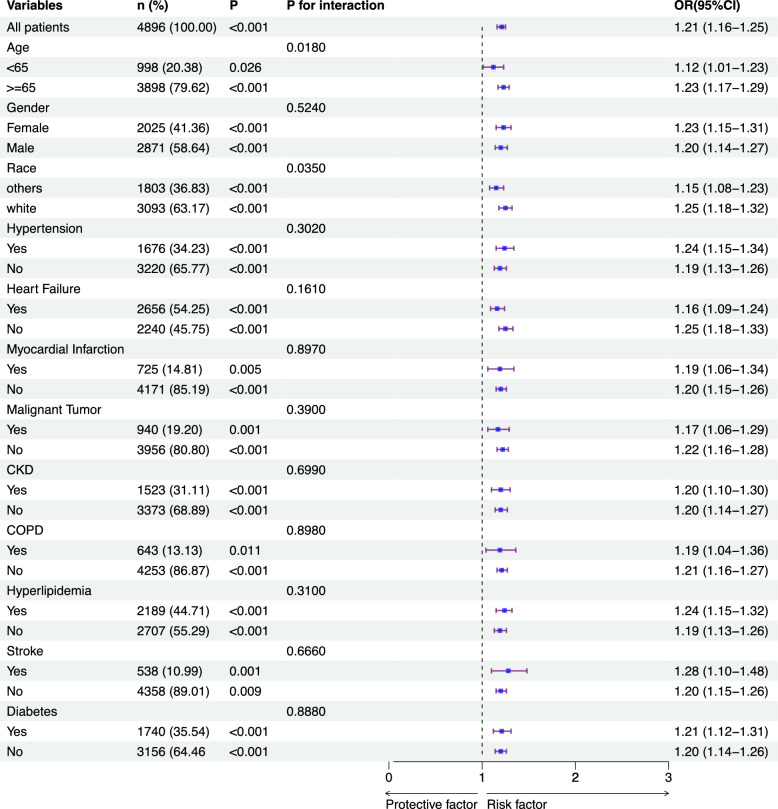
Forest plots of stratified analyses of EASIX and in-hospital all-cause mortality

Stratified analysis of subgroups revealed that the association between EASIX and in-hospital mortality was more pronounced in older patients (*P* < 0.001, *P* for interaction = 0.018) and those of White descent (*P* < 0.001, *P* for interaction = 0.035), as evidenced by higher OR in these groups. Specifically, the OR for older patients was 1.23 (95% CI 1.17–1.29), while for White patients, it was 1.25 (95% CI 1.18–1.32), indicating that both age and race significantly modify the effect of EASIX on in-hospital mortality (Fig. 7).

In the analysis of 28-day mortality, patients with a history of HF demonstrated a lower risk (*P* < 0.001, HR 1.13, 95% CI 1.09–1.18) compared to the control group (*P* < 0.001, HR 1.19, 95% CI 1.14–1.23) (*P* for interaction = 0.016).This finding suggests that the presence of HF may attenuate the effect of EASIX on 28-day mortality risk. Furthermore, no significant subgroup effects were observed in other strata, with all suggesting a significant association between EASIX and the outcome, further supporting the robustness of our conclusions (Supplementary Fig. S2).

When analyzing 365-day mortality, EASIX was significantly associated with mortality risk across all subgroups. However, significant interactions were observed for age (*P* for interaction = 0.010), race (*P* for interaction = 0.036), hypertension (*P* for interaction = 0.005), HF (*P* for interaction = 0.010), CKD (*P* for interaction = 0.003), and hyperlipidemia (*P* for interaction = 0.004), suggesting that these variables may modify the relationship between EASIX and long-term mortality risk (Supplementary Fig. S2).

## Discussion

As components of the EASIX calculation, LDH reflects both ED [24] and serves as a key marker of systemic inflammation [25]. In the event of inflammation and oxidative stress mediating endothelial cell necrosis or apoptosis [26], LDH is released from endothelial cells, leukocytes, and platelets, resulting in elevated plasma LDH levels [27]. Furthermore, inflammation and oxidative stress have been demonstrated to stimulate myocardial fibrosis, resulting in atrial structural remodeling and the induction of AF [28]. Furthermore, the reduced platelet count in the EASIX is indicative of endothelial injury and the activation of platelet adhesion and aggregation [29], processes initiated by vWF during vascular damage. vWF is significantly overexpressed in patients with AF [5], further exacerbating the risk of adverse cardiovascular events. Furthermore, given that EASIX also includes serum creatinine levels, elevated creatinine may reflect the connection between ED and renal impairment, as ED is a pathological basis in conditions, such as AKI [31] and diabetic nephropathy [32]. The present study corroborates the hypothesis that patients occupying the higher EASIX quartiles are more prone to developing AKI during the period of hospitalization, a factor which in turn contributes to elevated all-cause mortality (Supplementary Table S3).

Currently, ED as assessed by the Peripheral Arterial Reactivity Index has been established as a prognostic marker for atherosclerosis and cardiovascular events [33]. Baseline vascular ED assessed by RHI has been shown to predict 5-year recurrence and other cardiovascular events in patients with AF undergoing catheter ablation, allowing risk stratification [34]. Given that EASIX serves as a simple alternative for assessing ED and that its components reflect systemic inflammation, renal function and platelet aggregation status, this score has the potential to predict the risk of all-cause mortality in patients with AF. A study by the American Heart Association [35] highlights that despite the encouraging decrease in cardiovascular event-related mortality over the past decade, the overall prognosis for newly diagnosed AF patients has not improved due to the increase in non-cardiovascular deaths. Given the high incidence of AF in the ICU and the complex comorbidities of these patients, it is increasingly important to focus on the ED as a common pathway.

Recent advancements have been made in the treatment of ED through microRNA [36] and stem cells [37], but clinical translation remains filled with uncertainties and challenges. Consequently, given the prevalence of ED as a common pathway associated with various cardiovascular and metabolic diseases, a more prudent and feasible approach may be to explore the expansion of the indications of existing drugs. It has been established that antiplatelet drugs, such as Vorapaxar, can enhance nitric oxide release by regulating the protein kinase B (AKT) signaling pathway and intracellular calcium concentration, while also reducing cholesterol-induced DNA damage, thereby maintaining endothelial barrier integrity and promoting endothelial cell proliferation [38]. Ticagrelor has been shown to both reduce serum epidermal growth factor (EGF) levels and increase eNOS expression [39]. Antidiabetic drugs, such as metformin, have been observed to promote eNOS expression and enhance the microvascular structure of the femoral artery [40]. Empagliflozin, a sodium–glucose cotransporter 2 (SGLT2) inhibitor, has been demonstrated to activate AMP-activated protein kinase (AMPK), which in turn inhibits mitochondrial fission. This results in the preservation of cardiac microvascular barrier function and integrity, the maintenance of eNOS phosphorylation, and the improvement of microvascular density and perfusion. Large-scale trials have demonstrated that GLP-1 receptor agonists (GLP-1RAs) significantly reduce carotid intima–media thickness, thereby demonstrating anti-atherosclerotic properties and reducing the risk of cardiovascular events in diabetic patients [41]. The effects of these drugs on endothelial function may expand their therapeutic applications for patients with AF, though further studies are needed to explore this potential.

In the stratified analysis conducted, it was observed that the predictive value of EASIX for short- and long-term all-cause mortality risk was moderately influenced in patients with a history of HF, hypertension, and hyperlipidemia. This may be attributable to long-term pharmacological treatment, which has been demonstrated to enhance endothelial function and diminish the role of ED in the etiology of death. Despite the limitations imposed by data extraction, this study did not differentiate between new-onset and pre-existing AF. However, it is plausible that patients with new-onset AF, who have not yet undergone treatment, may benefit more from early monitoring of EASIX levels.

This study confirmed that EASIX is an independent predictor of both short-term and long-term all-cause mortality in critically ill patients with AF. The ROC analysis revealed that EASIX exhibited a comparable predictive performance to that of the SOFA, yet significantly outperformed the CHA2DS2–VASc score. The SOFA primarily focuses on organ failure in critically ill patients, especially those with septic shock [42]. Regardless of the presence of AF, when a patient experiences multiple organ failure, the prognosis is generally worsened. Therefore, although the SOFA lacks specificity for AF, it still demonstrated significant predictive value in this study. However, the SOFA involves a relatively complex scoring process, as it requires the assessment of multiple organ systems. In contrast, EASIX is simpler and more rapid, which could enhance clinical efficiency and streamline its application in practice. The CHA₂DS₂–VASc score primarily predicts the risk of stroke in patients with AF. Its components are all indicators that suggest the risk of vascular events, and it has been shown to effectively predict outcomes such as all-cause mortality in patients with AF undergoing coronary stenting [43] or in elderly patients with chronic heart failure (whether or not they have concomitant AF) [44]^.^ Beyond the cardiovascular system, previous studies have focused on predicting and stratifying the risk of mortality in patients with CKD [45] and those undergoing hemodialysis [46].Given the severe clinical condition of critically ill patients, the CHA₂DS₂–VASc score may lack the necessary monitoring markers beyond the cardiovascular system, which explains its relatively poor performance in predicting all-cause mortality in this study. In contrast, EASIX offers a simpler and more precise prognostic tool from the perspective of ED, providing meaningful predictive value for critically ill patients with AF.

Nevertheless, the present study is not without its limitations. First, as this study was conducted in a single center, there is a possibility of selection bias, and therefore, caution should be exercised when generalizing the findings. Second, the retrospective nature of the study may have resulted in the presence of residual confounding factors, despite our attempts to adjust for possible confounders. Third, EASIX was only assessed at ICU admission, and no further dynamic measurements were taken during the ICU stay. Future research should explore whether fluctuations in EASIX during hospitalization have clinical significance. Furthermore, the distinction between new-onset AF and pre-existing AF at the time of ICU admission was not made, nor was there a differentiation between paroxysmal and persistent AF. Further studies are needed to assess the applicability of our findings in these specific patient subsets. Finally, it is important to note that the parameters required to calculate EASIX are not specific to ED and should not be solely interpreted as indicators of ED in patients with AF. The absence of direct mechanistic studies precludes the ability to make causal inferences, and EASIX should be regarded as a prognostic indicator rather than a definitive causal marker for mortality in critically ill patients with AF. Notwithstanding the limitations of the present study, its findings contribute substantially to the development of suitable biomarkers derived from routine laboratory tests for the identification of high-risk patients. Hence, larger, multicenter studies are needed to further validate the role of EASIX as a prognostic marker in this population.

## Conclusion

The findings of this study indicate that EASIX is associated with in-hospital all-cause mortality in critically ill patients with AF, as well as with both short- and long-term all-cause mortality. This association remained significant even after adjusting for comorbidities and therapeutic interventions during ICU admission across different patient subgroups, demonstrating its robustness. ROC analysis further revealed that the prognostic performance of EASIX for both short- and long-term outcomes is comparable to that of the SOFA score. Consequently, it can be concluded that EASIX is a reliable and valuable indicator of poor prognosis in critically ill patients with AF.

## Supplementary Information

Additional file 1: Supplementary Table S1. ICD codes for diseases or comorbidities.

Additional file 2: Supplementary Table S2. Boruta algorithm and Lasso regression conducted the feature selection for the relationship between EASIX and in-hospital mortality.

Additional file 3: Supplementary Table S3. Baseline characteristics of patients based on the quartiles of the EASIX at admission.

Additional file 4: Supplementary Table S4. DeLong test for comparing the AUC of EASIX, SOFA, and CHA₂DS₂-VASc.

Additional file 5: Supplementary Table S5. Raw data on populations included in studies and analyses (after interpolation for missing).

Additional file 6: Supplementary Figure S1.

Additional file 7: Supplementary Figure S2.

## Data Availability

The original data for this study are publicly available from the MIMIC-IV database (Https://mimic.mit.edu) following registration and authentication. One of the authors of this paper, YX, has been granted authorization to use these de-identified data (Record ID: 59,051,976). The authors confirm that all data generated during the course of our study and analyses are included in the published article and its supplementary files. Further details may be obtained from the co-author, YX, upon reasonable request.
