# Supplementary material for: Risk analysis of the association between EASIX and all-cause mortality in critical ill patients with atrial fibrillation: a retrospective study from MIMIC-IV database
Source: Eur J Med Res. 2025 Apr 29;30:344. doi: 10.1186/s40001-025-02621-4 (PMC12039053; doi:10.1186/s40001-025-02621-4)
Supplement: Supplementary file 1 — Additional file 1: Supplementary Table S1. ICD codes for diseases or comorbidities. [file 40001_2025_2621_MOESM1_ESM.docx]

**Supplementary Table S1** ICD codes for diseases or comorbidities

| **Disease** | ICD-9 | ICD-10 |
| --- | --- | --- |
| Atrial fibrillation | 42731 | I4891,I480,I482,I4819,I4820,I481,I4821,I4811 |
| Hypertension | 4019, 4011, 4010 | I161, I10 |
| Heart Failure | 4280,42832,42822, 42833, 42823, 42830,42843,42831, ,42821,42842, 42820, 40491,40291,42841,4289,42840, 4281, 40201,40492 | I5032, I5033,I5022,I5023,I509, I5030, I5021,I5020, I5031,I5043,I5042, I5084,I50810,I5041,I5082,I5040, I50814,I50811,I50813,I50812, I5089,I5083 |
| Myocardial Infarction | 41000,41001,41002,41010,41011,41012,41020,41021,41022,41030,41031,41032,41040,41041,41042,41050,41051,41052,41080,41081,41082,41090,41091,41092 | I21,I219,I230,I231,I232,I233,I234,I235,I236,I238,I210,I2101,I2102,I2109,I211,I2111,I2119,I2121,I2129,I213,I214,I21A1,I21A9,I222 |
| Malignant Tumor | 1985, 1977,1970,1983, 185, 1976, 19889, 1629 | V103,V1046,Z85828,V1083,Z853, V1005, Z85038,Z800,V163,C787,C7951,V160,V1011,V1052,V1051, Z85118, Z8546, C786,C7931,Z803,C61,Z8551 |
| Chronic Kidney Disease | 40390,5859, 5853, 5854, 5852,40310, 5855 | I129,N189,N183, I130,I120, N184, N182, N185,E1122 |
| COPD | 49121,49120,4919, 4918, ,4910 | J42, J410, J449,J441,J440 |
| Hyperlipidemia | 2724, 2722 | E785,E7849,E782,E784 |
| Stroke | 431,43820,43811,4359, 43883,99702 | V1254,Z8673, V171,G459 |
| Diabetes |  |  |
| Type-1 | 25061,25001,25051,25041,25063,25013,25053,25043,25081 | E1022,E10319,E1065,E1040,E1043,E10649,E1010,E1021,E109,E1042,E1051,E10621 |
| Type-2 | 25000,25060,25040,25050,25002,25080,25062,25042,25082,25052,25070,25012,25092,25072,25090 | E119,E1122,E1165,E1140,E1151,E11319,E1142,E1121,E11649,E11621,E1169,E1143,E1152,E118,E11610,E11622,E1110,E11628,E1139,E1136 |
