# Supplementary material for: Risk analysis of the association between EASIX and all-cause mortality in critical ill patients with atrial fibrillation: a retrospective study from MIMIC-IV database
Source: Eur J Med Res. 2025 Apr 29;30:344. doi: 10.1186/s40001-025-02621-4 (PMC12039053; doi:10.1186/s40001-025-02621-4)
Supplement: Supplementary file 3 — Additional file 3: Supplementary Table S3. Baseline characteristics of patients based on the quartiles of the EASIX at admission. [file 40001_2025_2621_MOESM3_ESM.docx]

**Supplementary Table S3** Baseline characteristics of patients based on the quartiles of the EASIX at admission

| **Variables** | **Q1(<4.56)** | **Q2(4.56-5.64)** | **Q3(5.64-6.84)** | **Q4(>6.84)** | ***P*** |
| --- | --- | --- | --- | --- | --- |
|  |  |  |  |  |  |
| **Demographics** |  |  |  |  |  |
| Age,years | 74.00 (65.00,82.00) | 76.00 (68.00,84.00) | 76.00 (67.00,84.00) | 75.00 (66.00,82.25) | <0.001 |
| Male Gender, n(%) | 620 (50.65) | 690 (56.37) | 759 (62.01) | 802 (65.52) | <0.001 |
| Race, White, n(%) | 803 (65.60) | 764 (62.42) | 807 (65.93) | 719 (58.74) | <0.001 |
| Weight, kg | 77.70 (64.47,93.33) | 78.00 (65.99,95.00) | 81.20 (68.07,97.03) | 82.00 (69.10,98.00) | <0.001 |
| **Past history, n (%)** |  |  |  |  |  |
| Hypertension | 593 (48.45) | 464 (37.91) | 331 (27.04) | 288 (23.53) | <0.001 |
| Heart Failure | 472 (38.56) | 658 (53.76) | 779 (63.64) | 747 (61.03) | <0.001 |
| Myocardial Infarction | 91 (7.43) | 152 (12.42) | 209 (17.08) | 273 (22.30) | <0.001 |
| Malignant Tumor | 248 (20.26) | 227 (18.55) | 239 (19.53) | 226 (18.46) | 0.629 |
| Chronic Kidney Disease | 157 (12.83) | 321 (26.23) | 521 (42.57) | 524 (42.81) | <0.001 |
| COPD | 143 (11.68) | 172 (14.05) | 174 (14.22) | 154 (12.58) | 0.191 |
| Hyperlipidemia | 527 (43.06) | 577 (47.14) | 563 (46.00) | 522 (42.65) | 0.066 |
| Stroke | 139 (11.36) | 155 (12.66) | 121 (9.89) | 123 (10.05) | 0.098 |
| Diabetes | 341 (27.86) | 428 (34.97) | 479 (39.13) | 492 (40.20) | <0.001 |
| **Laboratory data** |  |  |  |  |  |
| WBC(10^9^/L) | 10.82 (8.02,14.90) | 10.98 (8.30,15.41) | 11.30 (8.07,16.05) | 11.87 (7.66,17.98) | **0.049** |
| RBC(10^9^/L) | 3.62 (3.16,4.13) | 3.51 (3.05,4.03) | 3.33 (2.90,3.87) | 3.21 (2.72,3.79) | <0.001 |
| Platelet(10^9^/L) | 245.38 (190.32,324.37) | 194.33 (151.00,255.12) | 169.33 (125.00,226.12) | 117.84 (70.88,174.92) | <0.001 |
| Hemoglobin(g/dL) | 10.65 (9.12,12.23) | 10.40 (9.00,11.90) | 9.95 (8.60,11.55) | 9.62 (8.20,11.27) | <0.001 |
| RDW(%) | 14.52 (13.50,16.07) | 14.93 (13.95,16.66) | 15.52 (14.25,17.30) | 16.24 (14.75,18.35) | <0.001 |
| Hct(%) | 32.55 (28.54,37.20) | 32.10 (28.08,36.53) | 30.54 (26.76,35.52) | 29.85 (25.59,34.95) | <0.001 |
| Sodium(mmol/L) | 138.50 (135.50,140.81) | 138.50 (136.00,141.00) | 138.33 (135.00,141.00) | 137.53 (134.00,141.50) | **0.003** |
| Potassium(mmol/L) | 4.00 (3.70,4.35) | 4.13 (3.82,4.50) | 4.28 (3.90,4.70) | 4.45 (4.00,5.00) | <0.001 |
| Calcium(mmol/L) | 8.40 (7.95,8.87) | 8.35 (7.90,8.85) | 8.37 (7.90,8.83) | 8.20 (7.67,8.70) | <0.001 |
| Chloride(mmol/L) | 103.00 (99.50,106.50) | 103.50 (99.38,107.33) | 103.25 (98.50,107.50) | 101.71 (97.00,106.50) | <0.001 |
| Glucose(mg/dL) | 123.00 (104.00,149.00) | 129.00 (107.88,161.27) | 134.00 (112.00,172.12) | 142.08 (113.65,189.00) | <0.001 |
| Anion gap(mmol/L) | 13.00 (11.33,15.00) | 14.00 (12.00,16.50) | 15.00 (12.63,17.33) | 17.00 (14.25,20.00) | <0.001 |
| PT(s) | 14.30 (12.70,16.90) | 15.10 (13.30,19.16) | 15.80 (13.53,21.66) | 18.20 (14.50,25.83) | <0.001 |
| PTT(s) | 32.10 (28.20,40.41) | 33.45 (28.85,44.74) | 35.83 (29.48,50.24) | 37.21 (30.20,53.05) | <0.001 |
| INR | 1.30 (1.15,1.55) | 1.40 (1.20,1.80) | 1.43 (1.23,2.00) | 1.70 (1.30,2.40) | <0.001 |
| Bilirubin(mg/dL) | 0.60 (0.40,0.85) | 0.70 (0.40,1.15) | 0.80 (0.50,1.35) | 1.00 (0.55,2.15) | <0.001 |
| ALT(U/L) | 19.00 (12.00,32.00) | 23.00 (15.00,42.00) | 28.00 (17.00,58.00) | 54.00 (23.00,241.18) | <0.001 |
| AST(U/L) | 25.00 (18.00,38.00) | 34.00 (22.00,60.62) | 46.84 (27.00,97.25) | 94.75 (40.00,444.50) | <0.001 |
| Urea nitrogen(mg/dL) | 15.75 (12.00,20.50) | 24.50 (18.33,34.54) | 38.00 (26.73,51.67) | 52.29 (35.31,78.00) | <0.001 |
| Serum creatinine(mg/dL) | 0.85 (0.68,1.05) | 1.13 (0.87,1.58) | 1.56 (1.10,2.35) | 2.23 (1.50,3.50) | <0.001 |
| LDH(U/L) | 210.00 (174.00,258.00) | 261.75 (212.75,338.00) | 320.00 (244.00,427.00) | 535.50 (340.00,1016.88) | <0.001 |
| **Vital signs** |  |  |  |  |  |
| Heart Rate(bpm) | 85.83 (74.14,99.04) | 85.16 (73.92,97.91) | 85.34 (74.26,98.05) | 88.73 (76.17,102.70) | <0.001 |
| systolic blood pressure(mmHg) | 115.30 (104.69,129.86) | 112.56 (103.14,125.67) | 109.58 (100.50,121.38) | 107.50 (99.96,118.13) | <0.001 |
| diastolic blood pressure(mmHg) | 65.50 (58.19,73.36) | 63.16 (56.27,70.92) | 61.22 (55.11,68.66) | 62.28 (55.38,69.10) | <0.001 |
| mean blood pressure(mmHg) | 78.68 (70.61,87.18) | 75.75 (69.32,84.21) | 73.46 (68.00,81.58) | 74.20 (67.99,81.13) | <0.001 |
| Respiratory Rate(bpm) | 19.32 (17.08,22.04) | 19.55 (17.31,22.11) | 19.76 (17.39,22.55) | 20.72 (18.00,23.94) | <0.001 |
| SpO2(%) | 96.80 (95.28,98.08) | 96.88 (95.43,98.20) | 96.81 (95.36,98.20) | 96.53 (95.03,97.97) | <0.001 |
| Temperature(℃) | 36.79 (36.62,37.03) | 36.81 (36.58,37.06) | 36.75 (36.55,37.03) | 36.74 (36.50,37.03) | <0.001 |
| **Medication, n (%)** |  |  |  |  |  |
| Glucocorticoids | 246 (20.10) | 293 (23.94) | 331 (27.04) | 422 (34.48) | <0.001 |
| ARB ACEI | 299 (24.43) | 349 (28.51) | 300 (24.51) | 183 (14.95) | <0.001 |
| Immunosuppressant | 23 (1.88) | 26 (2.12) | 34 (2.78) | 49 (4.00) | **0.005** |
| Aspirin | 581 (47.47) | 609 (49.75) | 624 (50.98) | 522 (42.65) | <0.001 |
| Statins | 172 (14.05) | 191 (15.60) | 171 (13.97) | 105 (8.58) | <0.001 |
| Beta blocker | 917 (74.92) | 926 (75.65) | 888 (72.55) | 806 (65.85) | <0.001 |
| Clopidogrel | 10 (0.82) | 15 (1.23) | 38 (3.10) | 33 (2.70) | <0.001 |
| Dipyridamo | 10 (0.82) | 13 (1.06) | 14 (1.14) | 10 (0.82) | 0.778 |
| Warfarin | 200 (16.34) | 261 (21.32) | 276 (22.55) | 202 (16.50) | <0.001 |
| Amiodarone | 246 (20.10) | 285 (23.28) | 322 (26.31) | 389 (31.78) | <0.001 |
| Digitalis | 98 (8.01) | 92 (7.52) | 100 (8.17) | 109 (8.91) | 0.654 |
| Diuretics | 709 (57.92) | 841 (68.71) | 885 (72.30) | 867 (70.83) | <0.001 |
| Norepinephrine | 334 (27.29) | 461 (37.66) | 635 (51.88) | 776 (63.40) | <0.001 |
| Phenylephrine | 390 (31.86) | 418 (34.15) | 465 (37.99) | 522 (42.65) | <0.001 |
| Vasopressin | 138 (11.27) | 195 (15.93) | 310 (25.33) | 464 (37.91) | <0.001 |
| Dopamine | 37 (3.02) | 53 (4.33) | 88 (7.19) | 104 (8.50) | <0.001 |
| Dobutamine | 24 (1.96) | 39 (3.19) | 99 (8.09) | 170 (13.89) | <0.001 |
| Epinephrine | 42 (3.43) | 88 (7.19) | 138 (11.27) | 183 (14.95) | <0.001 |
| **Intervention, n (%)** |  |  |  |  |  |
| Ventilation | 947 (77.37) | 1022 (83.50) | 1049 (85.70) | 1081 (88.32) | <0.001 |
| CRRT | 18 (1.47) | 67 (5.47) | 134 (10.95) | 283 (23.12) | <0.001 |
| **Length of stay (LOS), days** |  |  |  |  |  |
| LOS hospital, M (Q₁, Q₃) | 9.00 (5.59,15.08) | 9.33 (5.92,16.39) | 10.98 (6.49,18.49) | 11.68 (6.08,20.54) | <0.001 |
| LOS ICU, M (Q₁, Q₃) | 2.88 (1.81,5.12) | 3.13 (1.86,5.92) | 3.74 (2.07,7.40) | 3.98 (2.08,7.80) | <0.001 |
| **Evaluation scores** |  |  |  |  |  |
| SOFA, M (Q₁, Q₃) | 3.00 (1.00,5.00) | 5.00 (3.00,7.00) | 6.00 (4.00,9.00) | 8.00 (6.00,12.00) | <0.001 |
| OASIS, M (Q₁, Q₃) | 32.00 (27.00,38.00) | 34.00 (28.00,39.00) | 35.00 (29.00,41.00) | 37.00 (31.00,44.00) | <0.001 |
| APS III, M (Q₁, Q₃) | 40.00 (30.00,52.00) | 46.00 (36.00,61.00) | 54.00 (44.00,67.00) | 67.00 (52.00,82.00) | <0.001 |
| GCS, M (Q₁, Q₃) | 15.00 (14.00,15.00) | 15.00 (14.00,15.00) | 15.00 (14.00,15.00) | 15.00 (13.00,15.00) | **0.044** |
| SAPS II, M (Q₁, Q₃) | 35.00 (29.00,43.00) | 40.00 (33.00,49.00) | 44.00 (37.00,53.00) | 51.00 (42.75,62.00) | <0.001 |
| Charlson, M (Q₁, Q₃) | 6.00 (4.00,7.00) | 6.00 (5.00,8.00) | 7.00 (5.00,9.00) | 7.00 (5.00,9.00) | <0.001 |
| CHA2DS2 VASc, M (Q₁, Q₃) | 3.00 (2.00,4.00) | 4.00 (2.00,4.00) | 3.00 (2.00,4.00) | 3.00 (2.00,4.00) | <0.001 |
| **Events, n(%)** |  |  |  |  |  |
| AKI | 968 (79.08) | 1033 (84.40) | 1074 (87.75) | 1106 (90.36) | <0.001 |
| Sepsis | 653 (53.35) | 775 (63.32) | 878 (71.73) | 992 (81.05) | <0.001 |
| Is Hosp Dead | 150 (12.25) | 242 (19.77) | 333 (27.21) | 550 (44.93) | <0.001 |
| Death Within Hosp 28days | 191 (15.60) | 298 (24.35) | 361 (29.49) | 582 (47.55) | <0.001 |
| Death Within Hosp 90days | 300 (24.51) | 402 (32.84) | 510 (41.67) | 721 (58.91) | <0.001 |
| Death Within Hosp 180days | 359 (29.33) | 469 (38.32) | 574 (46.90) | 778 (63.56) | <0.001 |
| Death Within Hosp 365days | 405 (33.09) | 542 (44.28) | 662 (54.08) | 827 (67.57) | <0.001 |

Data are means ± SD, median (interquartile range), or n (%)

Abbreviations: WBC, white blood cell; RBC, red blood cell; RDW, red cell distribution width; Hct, hematocrit; PT, prothrombin time; PTT, partial prothrombin time; INR, international normalized ratio; ALT, alanine aminotransferase; AST, aspartate aminotransferase; LDH, lactate dehydrogenase; SpO2:oxyhemoglobin saturation; ACEIs/ARBs, angiotensin-converting enzyme inhibitors/angiotensin receptor blockers; CRRT, continuous renal replacement therapy; ICU, intensive care unit; SOFA, sequential organ failure assessment; APS III :SAPS II, simplified acute physiology score; OASIS, Oxford acute severity of illness score; GCS, Glasgow coma scale; Charlson, Charlson comorbidity index; EASIX, endothelial activation and stress index; AKI, acute kidney injury.
