# Supplementary material for: Risk analysis of the association between EASIX and all-cause mortality in critical ill patients with atrial fibrillation: a retrospective study from MIMIC-IV database
Source: Eur J Med Res. 2025 Apr 29;30:344. doi: 10.1186/s40001-025-02621-4 (PMC12039053; doi:10.1186/s40001-025-02621-4)
Supplement: Supplementary file 4 — Additional file 4: Supplementary Table S4. DeLong test for comparing the AUC of EASIX, SOFA, and CHA₂DS₂-VASc. [file 40001_2025_2621_MOESM4_ESM.docx]

**Table3 Delong test compares the AUC**

| **Scores** | **In-hospital mortality**  ***Z* *P*** | | **28-day mortality**  ***Z* *P*** | | **365-day mortality**  ***Z* *P*** |
| --- | --- | --- | --- | --- | --- |
| EASIX vs SOFA | -2.409 | 0.016 | -1.567 | 0.117 | 0.967 0.334 |
| EASIX vs CHA_2_DS_2_-VASc | 13.875 | ＜0.001 | 9.961 | ＜0.001 | 8.085 ＜0.001 |
