# Supplementary figures and images for: Risk analysis of the association between EASIX and all-cause mortality in critical ill patients with atrial fibrillation: a retrospective study from MIMIC-IV database
Source: Eur J Med Res. 2025 Apr 29;30:344. doi: 10.1186/s40001-025-02621-4 (PMC12039053; doi:10.1186/s40001-025-02621-4)

ROC :28-day all-cause mortality

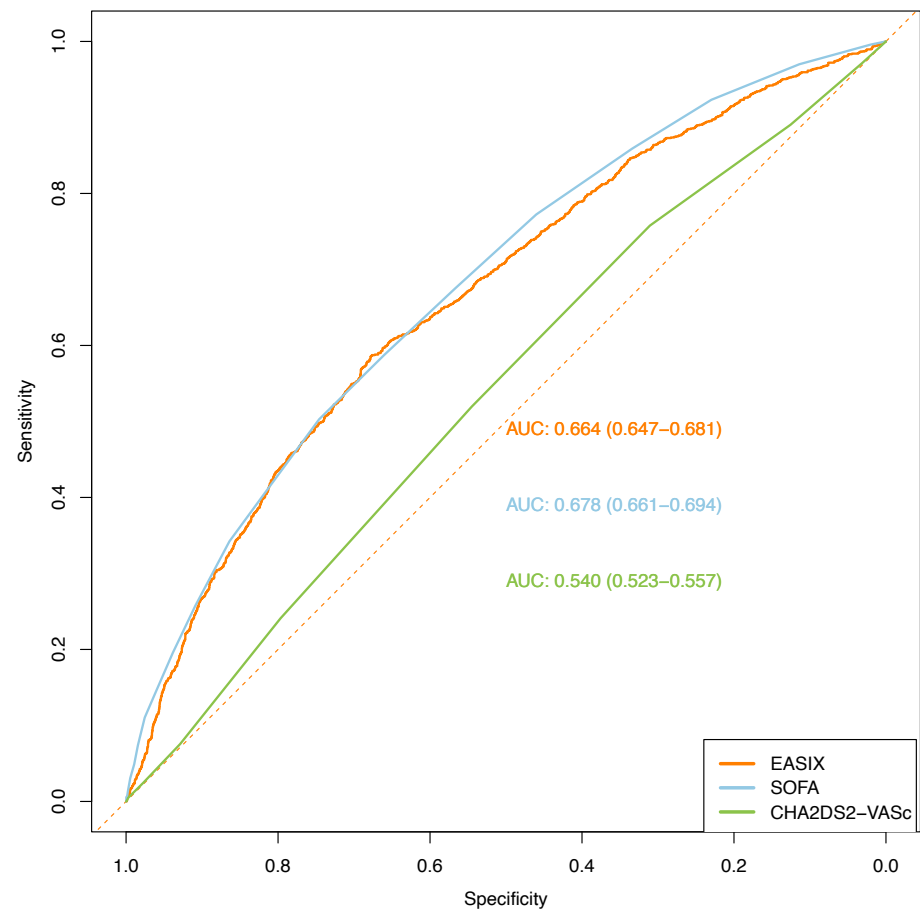

A

ROC :365-day all-cause mortality

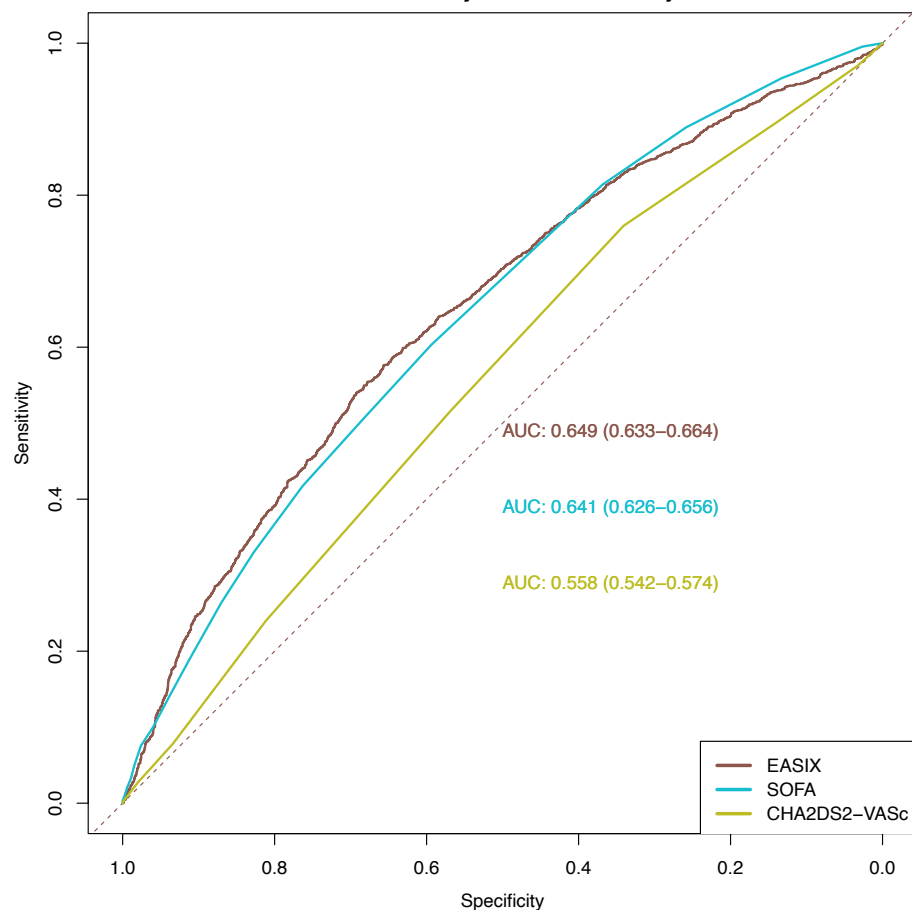

B

Supplement: Supplementary file 6 — Additional file 6: Supplementary Figure S1. [file 40001_2025_2621_MOESM6_ESM.pdf]
