# Supplementary material for: Risk analysis of the association between EASIX and all-cause mortality in critical ill patients with atrial fibrillation: a retrospective study from MIMIC-IV database
Source: Eur J Med Res. 2025 Apr 29;30:344. doi: 10.1186/s40001-025-02621-4 (PMC12039053; doi:10.1186/s40001-025-02621-4)
Supplement: Supplementary file 7 — Additional file 7: Supplementary Figure S2. [file 40001_2025_2621_MOESM7_ESM.pdf]

Variables	n (%)	P	P for interaction	HR(95%CI)
All patients	4896 (100.00)	<0.001		1.17 (1.13–1.20)
Age			0.2740	
<65	998 (20.38)	<0.001		1.12 (1.05–1.20)
>=65	3898 (79.62)	<0.001		1.18 (1.14–1.21)
Gender				
Female	2025 (41.36)	<0.001	0.7040	1.18 (1.13–1.23)
Male	2871 (58.64)	<0.001		1.17 (1.13–1.21)
Race			0.4280	
others	1803 (36.83)	<0.001		1.15 (1.10–1.21)
white	3093 (63.17)	<0.001		1.17 (1.13–1.22)
Hypertension			0.0540	
Yes	1676 (34.23)	<0.001		1.19 (1.13–1.25)
No	3220 (65.77)	<0.001		1.15 (1.11–1.19)
Heart Failure			0.0160	
Yes	2656 (54.25)	<0.001		1.13 (1.09–1.18)
No	2240 (45.75)	<0.001		1.19 (1.14–1.23)
Myocardial Infarction			0.1110	
Yes	725 (14.81)	<0.001		1.24 (1.16–1.34)
No	4171 (85.19)	<0.001		1.15 (1.12–1.18)
Malignant Tumor			0.1010	
Yes	940 (19.20)	<0.001		1.23 (1.16–1.30)
No	3956 (80.80)	<0.001		1.16 (1.12–1.19)
CKD			0.1040	
Yes	1523 (31.11)	<0.001		1.13 (1.08–1.19)
No	3373 (68.89)	<0.001		1.17 (1.14–1.21)
COPD			0.0790	
Yes	643 (13.13)	<0.001		1.25 (1.15–1.36)
No	4253 (86.87)	<0.001		1.16 (1.13–1.19)
Hyperlipidemia			0.0720	
Yes	2189 (44.71)	<0.001		1.18 (1.14–1.23)
No	2707 (55.29)	<0.001		1.15 (1.11–1.20)
Stroke			0.2510	
Yes	538 (10.99)	0.021		1.11 (1.02–1.21)
No	4358 (89.01)	<0.001		1.17 (1.14–1.21)
Diabetes			0.5270	
Yes	1740 (35.54)	<0.001		1.17 (1.11–1.22)
No	3156 (64.46.	<0.001		1.16 (1.13–1.20)

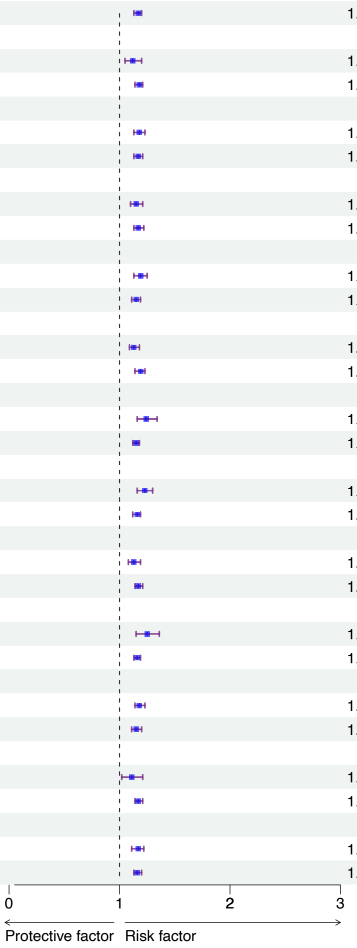

A

Forest plots of stratified analyses of EASIX and 28-day all-cause mortality

Variables	n (%)	P	P for interaction	HR(95%CI)
All patients	4896 (100.00)	<0.001		1.14 (1.12–1.17)
Age			0.0100	
<65	998 (20.38)	0.009		1.10 (1.04–1.15)
>=65	3898 (79.62)	<0.001		1.16 (1.13–1.19)
Gender			0.6810	
Female	2025 (41.36)	<0.001		1.16 (1.12–1.19)
Male	2871 (58.64)	<0.001		1.15 (1.11–1.18)
Race			0.0360	
others	1803 (36.83)	<0.001		1.11 (1.07–1.15)
white	3093 (63.17)	<0.001		1.16 (1.13–1.20)
Hypertension			0.0050	
Yes	1676 (34.23)	<0.001		1.17 (1.12–1.21)
No	3220 (65.77)	<0.001		1.12 (1.10–1.15)
Heart Failure			0.0100	
Yes	2656 (54.25)	<0.001		1.11 (1.08–1.15)
No	2240 (45.75)	<0.001		1.16 (1.12–1.19)
Myocardial Infarction			0.3940	
Yes	725 (14.81)	<0.001		1.19 (1.12–1.26)
No	4171 (85.19)	<0.001		1.14 (1.10–1.16)
Malignant Tumor			0.8150	
Yes	940 (19.20)	<0.001		1.14 (1.09–1.20)
No	3956 (80.80)	<0.001		1.15 (1.12–1.18)
CKD			0.0030	
Yes	1523 (31.11)	<0.001		1.10 (1.05–1.14)
No	3373 (68.89)	<0.001		1.15 (1.12–1.18)
COPD			0.4570	
Yes	643 (13.13)	<0.001		1.15 (1.07–1.22)
No	4253 (86.87)	<0.001		1.14 (1.12–1.17)
Hyperlipidemia			0.0040	
Yes	2189 (44.71)	<0.001		1.18 (1.14–1.22)
No	2707 (55.29)	<0.001		1.12 (1.09–1.15)
Stroke			0.3070	
Yes	538 (10.99)	<0.001		1.12 (1.05–1.20)
No	4358 (89.01)	<0.001		1.15 (1.12–1.17)
Diabetes			0.6550	
Yes	1740 (35.54)	<0.001		1.13 (1.09–1.17)
No	3156 (64.46.	<0.001		1.15 (1.12–1.18)

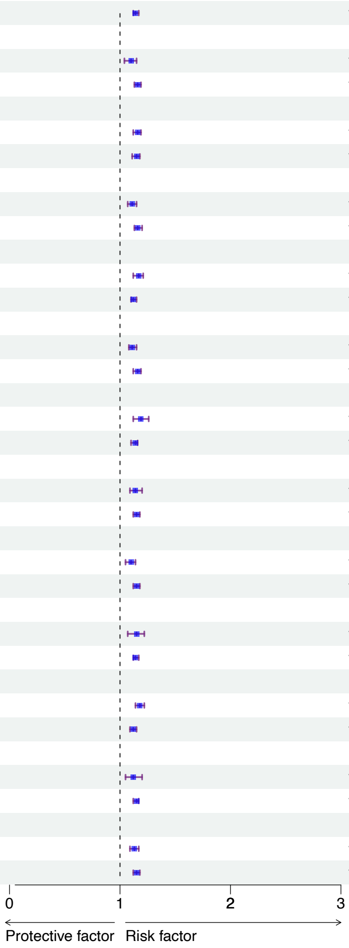

B

Forest plots of stratified analyses of EASIX and 365-day all-cause mortality
